# Equine Herpesvirus 1 Bridles T Lymphocytes To Reach Its Target Organs

**DOI:** 10.1128/JVI.02098-18

**Published:** 2019-03-21

**Authors:** Katrien C. K. Poelaert, Jolien Van Cleemput, Kathlyn Laval, Herman W. Favoreel, Liesbeth Couck, Wim Van den Broeck, Walid Azab, Hans J. Nauwynck

**Affiliations:** a Department of Virology, Immunology and Parasitology, Faculty of Veterinary Medicine, Ghent University, Merelbeke, Belgium; b Department of Molecular Biology, Princeton University, Princeton, New Jersey, USA; c Department of Morphology, Faculty of Veterinary Medicine, Ghent University, Merelbeke, Belgium; d Institut fur Virologie, Zentrum fur Infektionsmedezin, Freie Universitat Berlin, Berlin, Germany; Northwestern University

**Keywords:** Equidae, T lymphocytes, equine herpesvirus, immune evasion

## Abstract

Equine herpesvirus 1 (EHV1) replicates in the respiratory epithelium and disseminates through the body via a cell-associated viremia in leukocytes, despite the presence of neutralizing antibodies. “Hijacked” leukocytes, previously identified as monocytic cells and T lymphocytes, transmit EHV1 to endothelial cells of the endometrium or central nervous system, causing reproductive (abortigenic variants) or neurological (neurological variants) disorders. In the present study, we questioned the potential route of EHV1 infection of T lymphocytes and how EHV1 misuses T lymphocytes as a vehicle to reach the endothelium of the target organs in the absence or presence of immune surveillance. Viral replication was evaluated in activated and quiescent primary T lymphocytes, and the results demonstrated increased infection of activated versus quiescent, CD4^+^ versus CD8^+^, and blood- versus lymph node-derived T cells. Moreover, primarily infected respiratory epithelial cells and circulating monocytic cells efficiently transferred virions to T lymphocytes in the presence of neutralizing antibodies. Albeit T-lymphocytes express all classes of viral proteins early in infection, the expression of viral glycoproteins on their cell surface was restricted. In addition, the release of viral progeny was hampered, resulting in the accumulation of viral nucleocapsids in the T cell nucleus. During contact of infected T lymphocytes with endothelial cells, a late viral protein(s) orchestrates T cell polarization and synapse formation, followed by anterograde dynein-mediated transport and transfer of viral progeny to the engaged cell. This represents a sophisticated but efficient immune evasion strategy to allow transfer of progeny virus from T lymphocytes to adjacent target cells. These results demonstrate that T lymphocytes are susceptible to EHV1 infection and that cell-cell contact transmits infectious virus to and from T lymphocytes.

**IMPORTANCE** Equine herpesvirus 1 (EHV1) is an ancestral alphaherpesvirus that is related to herpes simplex virus 1 and causes respiratory, reproductive, and neurological disorders in Equidae. EHV1 is indisputably a master at exploiting leukocytes to reach its target organs, accordingly evading the host immunity. However, the role of T lymphocytes in cell-associated viremia remains poorly understood. Here we show that activated T lymphocytes efficiently become infected and support viral replication despite the presence of protective immunity. We demonstrate a restricted expression of viral proteins on the surfaces of infected T cells, which prevents immune recognition. In addition, we indicate a hampered release of progeny, which results in the accumulation of nucleocapsids in the T cell nucleus. Upon engagement with the target endothelium, late viral proteins orchestrate viral synapse formation and viral transfer to the contact cell. Our findings have significant implications for the understanding of EHV1 pathogenesis, which is essential for developing innovative therapies to prevent the devastating clinical symptoms of infection.

## Abstract (precis)

Equine herpesvirus 1 (EHV1) is an ancestral alphaherpesvirus that is related to herpes simplex virus 1 and causes respiratory, reproductive, and neurological disorders in Equidae. EHV1 is indisputably a master at exploiting leukocytes to reach its target organs, accordingly evading the host immunity. However, the role of T lymphocytes in cell-associated viremia remains poorly understood. Here we show that activated T lymphocytes efficiently become infected and support viral replication despite the presence of protective immunity. We demonstrate a restricted expression of viral proteins on the surfaces of infected T cells, which prevents immune recognition. In addition, we indicate a hampered release of progeny, which results in the accumulation of nucleocapsids in the T cell nucleus. Upon engagement with the target endothelium, late viral proteins orchestrate viral synapse formation and viral transfer to the contact cell. Our findings have significant implications for the understanding of EHV1 pathogenesis, which is essential for developing innovative therapies to prevent the devastating clinical symptoms of infection.

## INTRODUCTION

Herpesviruses are believed to have existed for over 300 million years and are associated with numerous infections among most species, including birds, fish, and mammals. Since their ancient origin, herpesviruses have efficiently coevolved with their hosts. The family *Herpesviridae* is divided into three subfamilies, *Alpha*-, *Beta*-, and *Gammaherpesvirinae*, based on common features and structures, such as genomic sequence arrangements (1). In the phylogenetic reconstruction of herpesviruses, equine herpesvirus 1 (EHV1) stands out as one of the most ancestral of all alphaherpesviruses (2). EHV1 is closely related to the ubiquitous human herpesviruses, such as herpes simplex virus 1 (HSV-1) and varicella zoster virus (VZV), making it an ideal model for studying the pathogenesis of alphaherpesviruses in general (2).

EHV1 is a highly prevalent pathogen in horse populations worldwide, since the majority of horses (>60%) are latently infected (3, 4). Horses become infected within the first year of life, and this cannot be inhibited by current vaccines (3). Notwithstanding the endemic state of EHV1 in equine populations, clinical outbreaks can occur, causing extensive economic consequences through treatment, biosafety procedures, and loss of performance (3, 5).

EHV1 can spread via respiratory secretions during (in)direct contact between horses. After primary replication in the epithelium of the upper respiratory tract (URT), EHV1 crosses the basement membrane and enters the bloodstream in individual infected leukocytes (6, 7). EHV1 has evolved the ability to enter, infect, and exploit monocytic (CD172a^+^) cells by T and B lymphocytes to reach the endothelium of the pregnant uterus or central nervous system (CNS) while evading the immune surveillance. Infection of the target endothelium might result in thromboembolic disease and ischemia, ultimately causing neonatal foal death, late-term abortion, or myeloencephalopathy (8–12).

Abortigenic and neurovirulent EHV1 phenotypes circulating in the field can be distinguished based on a single nucleotide polymorphism in the viral DNA polymerase gene (13). During a neurovirulent EHV1 respiratory infection, EHV1 mainly orchestrates the recruitment of CD172a^+^ monocytic cells from the blood circulation to the respiratory mucosa via the upregulation of specific chemokines, such as chemokine ligand 2 (CCL2) and CCL5 (14). In contrast, abortigenic EHV1 variants replicate in the respiratory epithelium while limiting the recruitment of immune cells to the URT (6, 14). Interestingly, Laval et al. (9) demonstrated that the gene expression of abortigenic EHV1 strains in CD172a^+^ cells is silenced and tightly regulated by histone deacetylases (HDAC). However, no delayed viral replication was observed in monocytic CD172a^+^ cells following infection with neurovirulent EHV1 variants (15). These findings suggested that abortigenic and neurovirulent EHV1 strains use distinct immune evasive strategies to persist in the host. While EHV1 infection of monocytic CD172a^+^ cells is widely described, little information is available on the role of lymphocytes in the pathogenesis of EHV1. In the URT, resident T lymphocytes and extravasated circulating T cell subsets patrol and mediate a rapid and protective immune reaction to suppress viral replication and spread (16–21). Despite the protective antiviral effects of T lymphocytes in the respiratory mucosa, EHV1 demonstrates a propensity for T lymphocytes. Indeed, CD8^+^ T lymphocytes are defined as a predominant site of EHV1 latency (22–24). However, it remains unclear how T lymphocytes at the respiratory mucosa and local lymphoid tissues become primarily infected. So far, van der Meulen et al. (8) have demonstrated the ability of blood-derived T lymphocytes to support infection with the abortigenic EHV1 strain 97P70 *in vitro*. They showed that EHV1 is mainly T lymphotropic upon activation of lymphocytes with specific mitogens. Mitogens trigger the activation of the mitogen-activated protein (MAP) kinase pathway. This signaling pathway is similarly activated by the interaction of the T cell receptor (TCR) and CD3 complex with a viral antigen, presented by the major histocompatibility complex (MHC) of an antigen-presenting cell (APC) (25, 26). Activated T lymphocytes predominantly produce large amounts of interleukin-2 (IL-2), a prototypical T-lymphocyte growth factor, which promotes allogeneic T cell activation and proliferation (27, 28). It is well known that activated T cells are more susceptible to HSV-1, VZV, and bovine herpesvirus 1 (BoHV1) infections (29–31). Comparably, the activation of equine T lymphocytes increases the number of EHV1-positive cells 4- to 12-fold, depending on the stimulating mitogen (8, 21, 32).

Infecting the cellular arm of the immune response helps EHV1 to suppress elimination by the immune system and allows viral spread within the host despite the presence of neutralizing antibodies. In addition, infectious virus can also be transmitted to other cells via cell-to-cell contact, allowing the virus to remain under the radar of the immune system (30, 33–35). Several studies have already demonstrated the potential of cell-free EHV1 to infect monocytes and T lymphocytes *in vitro* (6, 8, 9). However, any differences in susceptibility of T lymphocytes to EHV1 infection and subsequent cell-to-cell transfer mechanisms are still unclear. In this study, we determined whether abortigenic and neurovirulent EHV1 variants can directly infect and replicate in circulating and/or respiratory resident T lymphocytes or whether the virus first enters monocytic cells and/or the epithelium of the URT, followed by cell-to-cell transfer of virus particles to T lymphocytes. Next, we examined which T cell subpopulation is more susceptible to EHV1 infection and whether/how EHV1-infected T lymphocytes can transfer infection to the target endothelium in the presence of the immune response as an important step toward secondary replication of the virus.

## RESULTS

### EHV1 directly infects blood- and lymph node-derived T lymphocytes.

T lymphocytes derived from blood and pulmonary lymph nodes were inoculated with two abortigenic (97P70 and 94P247) and two neurovirulent (03P37 and 95P105) EHV1 strains. At 1, 3, 6, 9, 12, and 24 h postinfection (hpi), T lymphocytes and supernatant were collected for immunofluorescence staining and virus titration to determine intracellular and extracellular virus titers.

**FIG 1:**
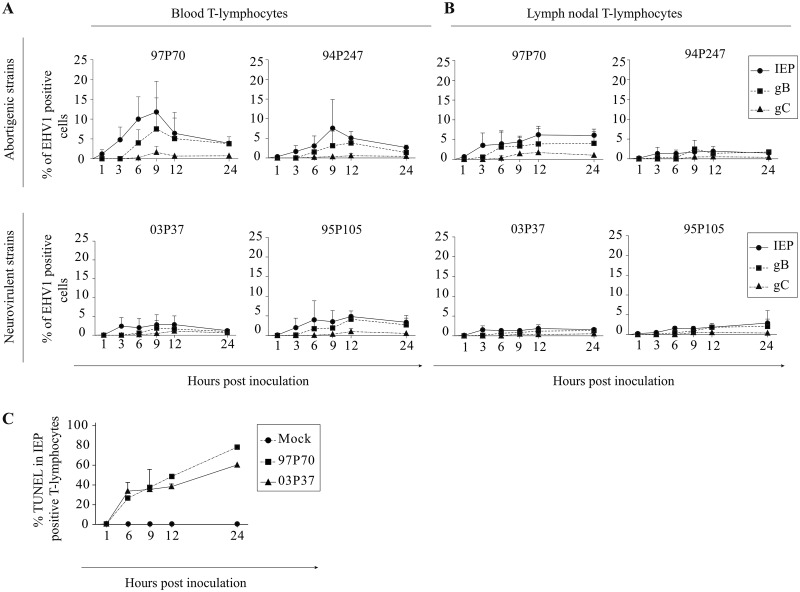
Replication kinetics of abortigenic (upper panels) and neurovirulent (lower panels) EHV1 variants in (A) blood-derived and (B) lymph node-derived T lymphocytes. The expression of immediate early proteins (IEP), leaky late glycoprotein B (gB) proteins, and real late glycoprotein C (gC) proteins was analyzed by immunofluorescence staining. (C) Cell viability of EHV1-infected cells was examined by double immunofluorescence staining by analyzing double IEP- and TUNEL-positive cells. Data represent means plus SD for three independent experiments.

In approximately 0.5% of the blood-derived T lymphocytes, immediate early protein (IEP) was first detected at 1 hpi with all EHV1 strains (Fig. 1A). The percentage of IEP-positive cells increased over time for both abortigenic strains, to 7% ± 7% (97P70) and 4% ± 3% (94P247) at 6 hpi (Fig. 1A, upper panel). Similarly, for the neurovirulent strains, 2% ± 2% (03P37) and 4% ± 4% (95P205) of the T lymphocytes became IEP positive at 6 hpi (Fig. 1A, lower panel). T lymphocytes inoculated with the abortigenic strains reached a maximum IEP expression of 10% ± 12% (97P70) and 8% ± 7% (94P247) at 9 hpi. The percentage of IEP-positive T cells upon inoculation with the neurovirulent variants increased from 3% ± 2% (03P37) and 3% ± 3% (95P105) at 9 hpi and reached a maximum of 3% ± 2% (03P37) and 5% ± 1% (95P105) IEP-positive cells at 12 hpi. Subsequently, the expression of IEP declined for both types of variants, to 3% ± 2% (97P70), 3% ± 1% (94P247), 3% ± 2% (03P37), and 3% ± 1% (95P105) at 24 hpi.

Similarly, the number of IEP-positive lymph nodal T cells increased from 1 to 6 hpi, from 0.5% to 2% for both abortigenic strains (Fig. 1B, upper panel) and from 0.2% to 1% for both neurovirulent strains (Fig. 1B, lower panel). The percentage of EHV1-inoculated lymph nodal T lymphocytes reached a maximum of 6% ± 1% (97P70) and 2% ± 1% (94P247) at 12 hpi. Both neurovirulent strains reached a maximum of 2% ± 1% (03P37) and 3% ± 3% (95P105) at 24 hpi. In both T cell populations, “leaky” late gB and late gC proteins were first detected at 3 and 6 hpi, respectively. The percentages of gB- and gC-positive blood-derived T cells reached maximum values of 7% ± 8% and 1% ± 2% (97P70), respectively, and 4% ± 1% and 1% ± 0.3% (94P247), respectively, at 9 hpi (abortigenic) and 2% ± 1% and 1% ± 1% (03P37), respectively, and 4% ± 1% and 1% ± 1% (94P247), respectively, at 12 hpi (neurovirulent). Similarly, for lymph node-derived T cells, we detected maximum values for gB- and gC-positive cells of 4% ± 1% and 2% ± 1% (97P70), respectively, and 2% ± 1% and 1% ± 0% (94P247), respectively, at 9 hpi (abortigenic) and 1% ± 1% and 1% ± 0% (03P37), respectively, and 2% ± 1% and 1% ± 0% (95P105), respectively, at 12 hpi (neurovirulent). Comparably to the expression of IEP, decreases in the percentages of gB- and gC-positive T cells were observed at 12 hpi.

Thus, all EHV1 strains showed similar replication kinetics in both T cell populations. However, at 9 hpi, significantly more blood-derived T cells were IEP positive upon inoculation with the 97P70 strain than upon inoculation with the 03P37 and 95P105 strains (*P* < 0.001 and *P* < 0.05, respectively). Comparable results were observed in the lymph nodal T lymphocytes. These results suggest differences in the T cell tropism of the circulating EHV1 strains, which can be interpreted as virulence differences between the strains. Furthermore, we found that blood-derived T cells are significantly more susceptible than lymph node-derived T cells to infection with cell-free EHV1 (*P* < 0.05).

To explain the decreased number of EHV1-infected T lymphocytes between 9 and 24 hpi, EHV1-positive apoptotic cells were visualized by terminal deoxynucleotidyltransferase-mediated dUTP-biotin nick end labeling (TUNEL) as demonstrated in Fig. 1C. At 1 hpi, the percentage of EHV1-positive apoptotic cells remained lower than 1%. The number of EHV1-positive T lymphocytes that became TUNEL positive increased significantly over time for both EHV1 strains. At 24 hpi, 76.0% ± 12.0% of 97P70-positive and 59.0% ± 2.0% of 03P37-positive T lymphocytes went into apoptosis.

Taking all these observations together, we conclude that blood- and lymph node-derived T lymphocytes are susceptible to both abortigenic and neurovirulent EHV1 variants. A faster and distinct replication kinetics was detected for both abortigenic strains in blood-derived T lymphocytes compared to that of the lymph node-derived T lymphocytes and the neurovirulent phenotypes. We detected programmed cell death in the EHV1-infected T lymphocytes early in infection (6 hpi), resulting in a decrease of EHV-positive T lymphocytes.

### IL-2-mediated stimulation of T lymphocytes enhances susceptibility to EHV1 infection.

Since blood-derived T lymphocytes were more sensitive to an EHV1 infection and no significant differences were observed between the EHV1 strains of the same phenotype, all subsequent experiments were performed with blood-derived T lymphocytes inoculated with one abortigenic (97P70) and one neurovirulent (03P37) EHV1 strain, unless stated differently.

**FIG 2:**
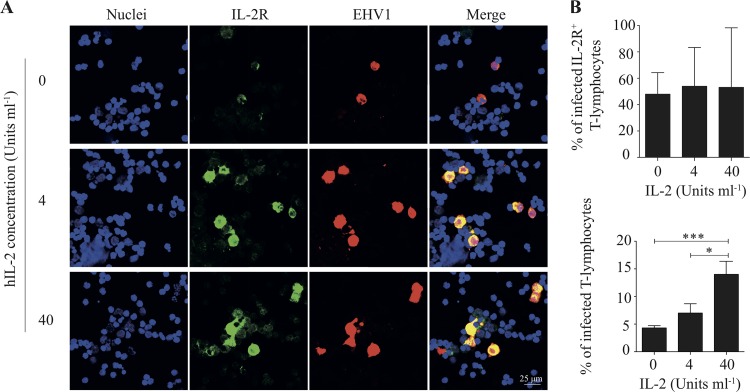
Recombinant interleukin-2 (IL-2) enhances T-lymphocyte infectivity. Primary T lymphocytes were stimulated overnight with 0, 4, or 40 U ml^−1^ of IL-2. (A) T cell activation was confirmed by double immunofluorescence staining. The IL-2 receptor (IL-2R) was stained green, and the EHV1 proteins were stained red. Nuclei were counterstained blue (Hoechst dye). (B) (Top) Upon overnight IL-2 stimulation and mock/EHV1 inoculation, the percentage of IL-2R expression was calculated and graphed. (Bottom) Overnight IL-2 stimulation of T lymphocytes increased the percentage of EHV1-positive T lymphocytes in a concentration-dependent manner. Data represent means plus SD for three independent experiments. *, *P* < 0.05; ***, *P* < 0.001.

The study of van der Meulen et al. (8) reported that equine blood T lymphocytes become more permissive to EHV1 replication once these cells are activated. The effect of T-lymphocyte activation was confirmed in the present study by overnight incubation with different concentrations of human interleukin-2 (IL-2) (0, 4, and 40 U ml^−1^). The combination of IL-2 stimulation and EHV1 inoculation activated the T cells, as visualized by double immunofluorescence staining of the IL-2 receptor (IL-2R) and of EHV1 proteins (Fig. 2A). Upregulation of the IL-2R is known to be a reliable marker of T cell activation and proliferation (36–38). The percentage of IL-2R expression (i.e., region of interest [ROI]) was calculated. In untreated T lymphocytes, 0.4% ± 0.4% of the mock-inoculated T cells and 2.7% ± 0.4% of the EHV1-inoculated T cells expressed detectable levels of the IL-2R. Pretreatment of the T lymphocytes with 4 and 40 U ml^−1^ IL-2 led to significant increases of this percentage, to 22.4% ± 0.9% (*P* < 0.001) and 22.2% ± 0.3% (*P* < 0.001), respectively, in the mock-inoculated T cells and 31.7% ± 5.0% (*P* < 0.001) and 26.0% ± 2.3% (*P* < 0.001), respectively, in the EHV1-inoculated T lymphocytes at 9 hpi. Interestingly, a significant correlation between EHV1 infection and the expression of IL-2R was observed (*P* < 0.01) at 9 hpi. All EHV1-positive cells were IL-2R positive. However, not all T lymphocytes that expressed IL-2R were EHV1 positive (Fig. 2B, top panel). Increasing the IL-2 concentration significantly amplified the percentage of EHV1-positive T cells, from 3.8% ± 0.9% at 0 U ml^−1^ to 12.8% ± 3.0% at 40 U ml^−1^ human IL-2 (hIL-2) (Fig. 2B, bottom panel). Statistically significant differences were observed between 0 and 40 U ml^−1^ (*P* < 0.001) and between 4 and 40 U ml^−1^ (*P* < 0.05).

These findings demonstrate that the important physiological immune mediator IL-2 efficiently activates T lymphocytes. *In vivo* IL-2 is produced in the context of the intimate interaction of antigen-presenting cells and T lymphocytes (39). On mimicking of this *in vivo* interaction by pretreatment with IL-2, T lymphocytes become more susceptible to EHV1 infection.

### EHV1 predominantly infects CD4^+^ T lymphocytes.

**FIG 3:**
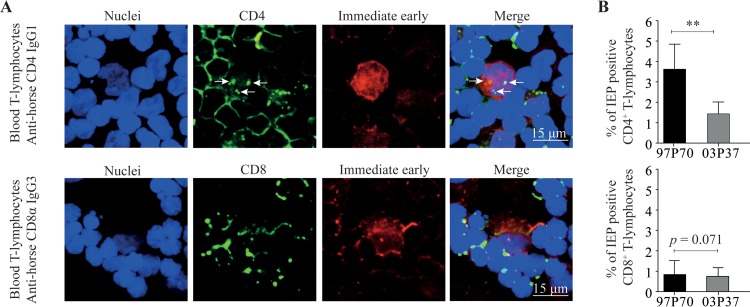
CD4^+^ and CD8^+^ subsets of T lymphocytes become infected with EHV1. Both T cell subsets were isolated by positive MACS separation and inoculated with the 97P70 or 03P37 EHV1 strain. (A) At 9 hpi, cells were collected and then subjected to double immunofluorescence staining for the CD4/CD8 cell marker (green) and viral IEP (red). Nuclei were counterstained blue (Hoechst dye). Arrows indicate clustered CD4. (B) Percentages of IEP-positive CD4^+^ and CD8^+^ T lymphocytes. All experiments were performed with blood-derived T lymphocytes from three different horses. Data represent means plus SD.

Next, we determined which specific subsets of T lymphocytes are more susceptible to EHV1 infection. We selected CD4^+^ and CD8^+^ blood T lymphocytes by positive magnetically activated cell sorting (MACS), followed by inoculation with an abortigenic (97P70) or neurovirulent (03P37) EHV1 strain. We detected IEP-positive CD4^+^ and CD8^+^ T lymphocytes by confocal microscopy (Fig. 3A). The expression of CD4 in the IEP-positive T lymphocytes was clustered into small patches, indicating T cell activation (40). The expression of CD8 consists of one patched signal. At 9 hpi, the CD4^+^ T cell subset was significantly more infected with both EHV1 variants than the CD8^+^ cells (*P* < 0.001). IEP was expressed in 3.6% ± 1.2% of EHV1-97P70- and 1.4% ± 0.6% of EHV1-03P37-inoculated CD4^+^ T lymphocytes. Abortigenic EHV1 strain 97P70 replication in CD4^+^ cells was more efficient than that of the neurovirulent 03P37 strain (*P* < 0.01). However, a small percentage of CD8^+^ T lymphocytes showed an IEP signal (0.8% ± 0.7% for 97P70 and 0.7% ± 0.4% for 03P37) (*P* = 0.072) (Fig. 3B).

Our results demonstrate that both EHV1 variants infect CD4^+^ and CD8^+^ T lymphocytes. We demonstrated that EHV1 preferentially infects CD4^+^ over CD8^+^ T cells. The abortigenic variant used in this study was more efficient than the neurovirulent variant at infecting CD4^+^ T lymphocytes.

### The multiplicity of infection impacts the percentage of EHV1^+^ T lymphocytes.

**FIG 4:**
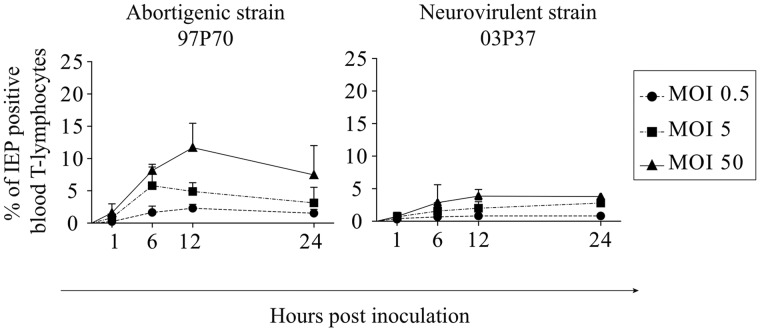
Multiplicity of infection affects the percentage of EHV1^+^ T lymphocytes. Blood T lymphocytes were inoculated at an MOI of 0.5, 5, or 50 with the abortigenic 97P70 (left panel) or neurovirulent 03P37 (right panel) EHV1 strain. Percentages of IEP-positive T cells are shown. Data represent means plus SD for experiments performed in triplicate.

We analyzed whether EHV1 infection of T lymphocytes is dependent on the multiplicity of infection (MOI). We inoculated blood-derived T lymphocytes with EHV1 strains 97P70 and 03P37 at different MOI (0.5, 5, and 50). Increasing the MOI significantly altered the number of IEP-positive cells at 6 hpi for the abortigenic strain (Fig. 4, left panel). The increase of the MOI from 0.5 to 50 significantly increased the percentage of IEP-positive cells at 6 hpi, from 1.7% ± 1.0% to 8.1% ± 1.0% (*P* < 0.05). The percentage of positive cells reached a plateau at an MOI of 5 for the EHV1-97P70 strain. Indeed, increasing the MOI from 5 to 50 did not significantly increase the percentage of IEP-positive T lymphocytes (5.8% ± 2.9% and 8.1% ± 1.0%) (*P* = 0.370). Similar results were observed at 12 (*P* = 0.310) and 24 (*P* < 0.05) hpi for increasing the MOI from 0.5 to 5 for the 03P37 strain (Fig. 4, right panel). Elevating the MOI from 5 to 50 showed a slight but not significant trend at 12 hpi (*P* = 0.091). The percentage of EHV1-positive T lymphocytes was significantly lower for the neurovirulent EHV1 strain than for the abortigenic variant, independent of the MOI used.

Taking these data together, we conclude that EHV1 infection of T lymphocytes is MOI dependent. The maximum percentage of EHV1-positive T lymphocytes was reached upon inoculation at an MOI of 5.

### Spatiotemporal distribution dynamics of EHV1 proteins in T lymphocytes.

**FIG 5:**
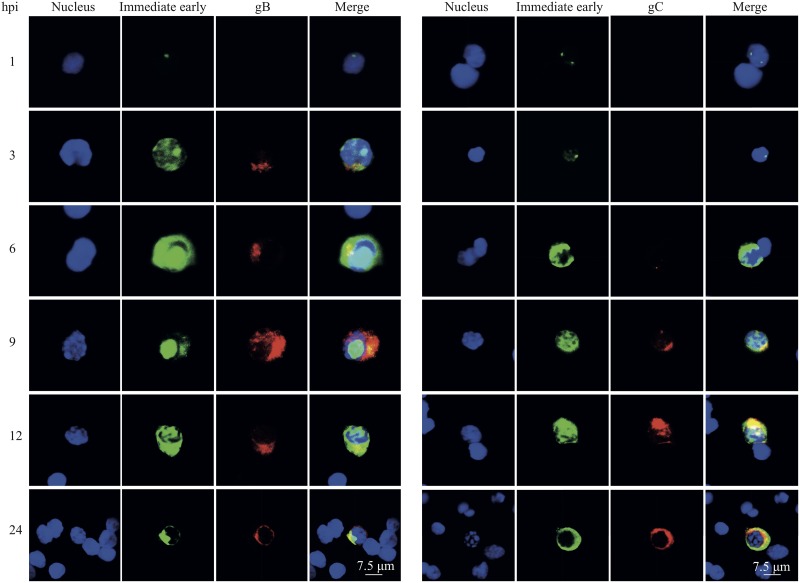
Spatiotemporal distribution of immediate early protein (IEP), leaky late glycoprotein B (gB) (left panel), and real late glycoprotein C (gC) (right panel) in blood-derived T lymphocytes. IEP was stained green, and gB and gC were visualized in red. Nuclei were counterstained blue (Hoechst dye).

Next, we analyzed the distribution of IEP and the leaky late gB and late gC proteins by immunofluorescence staining of infected T lymphocytes. Since no differences in the distribution of viral proteins in the cell were observed between the abortigenic and neurovirulent phenotypes, only confocal images of the abortigenic EHV1 strain 97P70 are shown in Fig. 5. The expression of IEP in EHV1-inoculated T lymphocytes was first detected at 1 hpi, with one or two foci of intranuclear staining. The intensity of IEP increased and the signal became more diffuse at 3 hpi. At this time point, leaky late gB proteins were found to be weakly expressed exclusively in the cytoplasm. At 6 hpi, IEP assembled together in nucleus-associated compartments, which occupied most of the nuclear space. The expression of gB proteins intensified during the course of infection. The IEP signal remained globular at 9 hpi. The late gC protein signal was detected at 6 hpi and increased as infection progressed. At 12 and 24 hpi, the size of the IEP globular structure decreased, and the IEP signal was distributed over the entire nucleus. The gB and gC signals were steadily present throughout the course of infection.

Taken together, these data show that EHV1 efficiently replicates in T lymphocytes. A fast and dexterous replication is observed in T lymphocytes, with the expression of all classes of viral proteins.

### Membrane expression of viral proteins.

**FIG 6:**
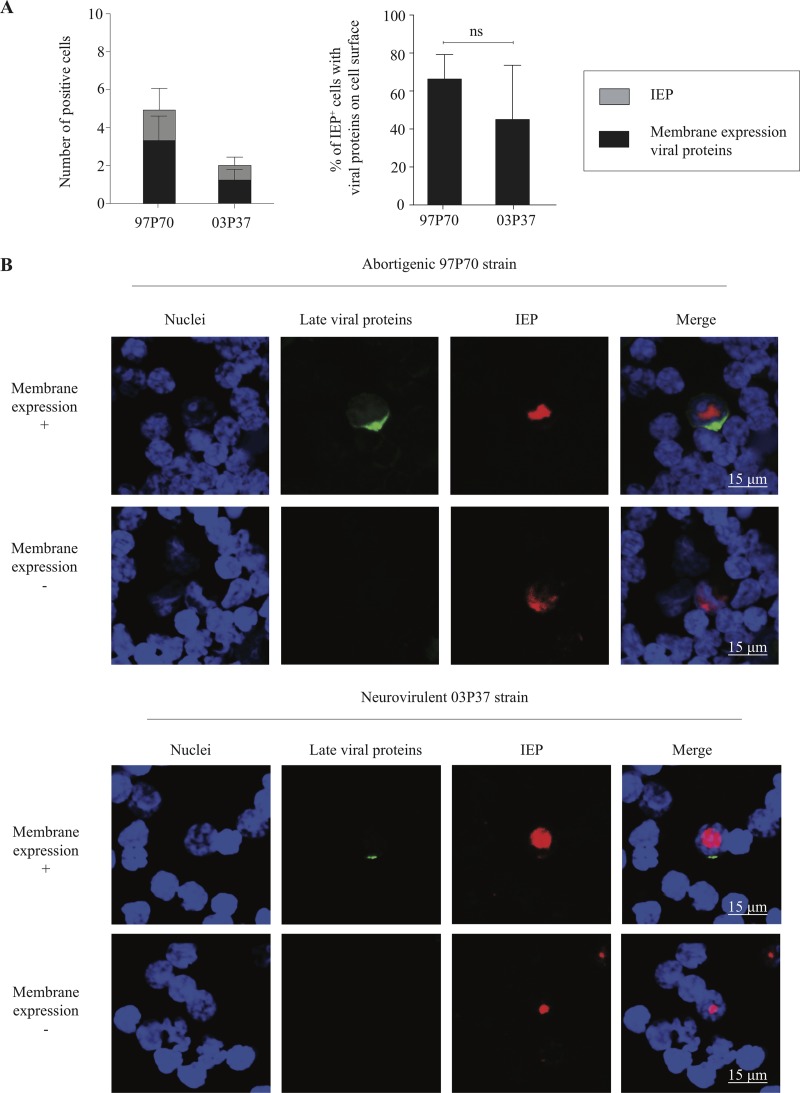
Viral glycoproteins are expressed on the plasma membrane for the majority of infected T lymphocytes. (A) T lymphocytes inoculated with the abortigenic 97P70 or neurovirulent 03P37 EHV1 strain were fixed at 9 hpi. Membrane expression of viral glycoproteins and the expression of IEP were analyzed. (B) Double immunofluorescence staining of viral glycoproteins on the cell surface (green) and of IEP (red). Nuclei were counterstained blue (Hoechst dye). Data represent means plus SD for three independent experiments. ns, no statistically significant differences.

To examine membrane expression of viral glycoproteins on the plasma membrane of infected T lymphocytes, abortigenic and neurovirulent EHV1-inoculated T lymphocytes were collected at 9 hpi. Figure 6 shows the percentages of IEP-positive T lymphocytes expressing viral proteins on the cell surface and representative confocal images. At 9 hpi, 5.0% ± 1.1% of the abortigenic 97P70-inoculated T lymphocytes became IEP positive, and 3.3% ± 1.3% of the T cells expressed viral proteins on the cell surface. For the neurovirulent 03P37 strain, 2.2% ± 0.1% of the T lymphocytes became IEP positive, and 1.1% ± 0.5% of the cells expressed viral proteins on the plasma membrane (Fig. 6A, left panel). No significant differences in membrane expression were observed between both EHV1 variants (*P* = 0.304) (Fig. 6A, right panel). Remarkably, when viral glycoproteins were detected on the cell surface, they were distributed into large aggregates to one side of the cell rather than having a homogeneous distribution over the plasma membrane (Fig. 6B, upper panel). The lower panel of Fig. 6B shows EHV1-positive T lymphocytes lacking viral glycoproteins on the plasma membrane.

Taken together, these data show that the majority of EHV1-infected T lymphocytes express viral proteins on the cell surface for both EHV1 variants, indicating full viral replication in T lymphocytes. Viral glycoproteins on the cell membrane aggregate into large patches on one side of the cell, suggesting the sequestration of viral proteins on the plasma membrane.

### Kinetics of EHV1 production in T lymphocytes.

**FIG 7:**
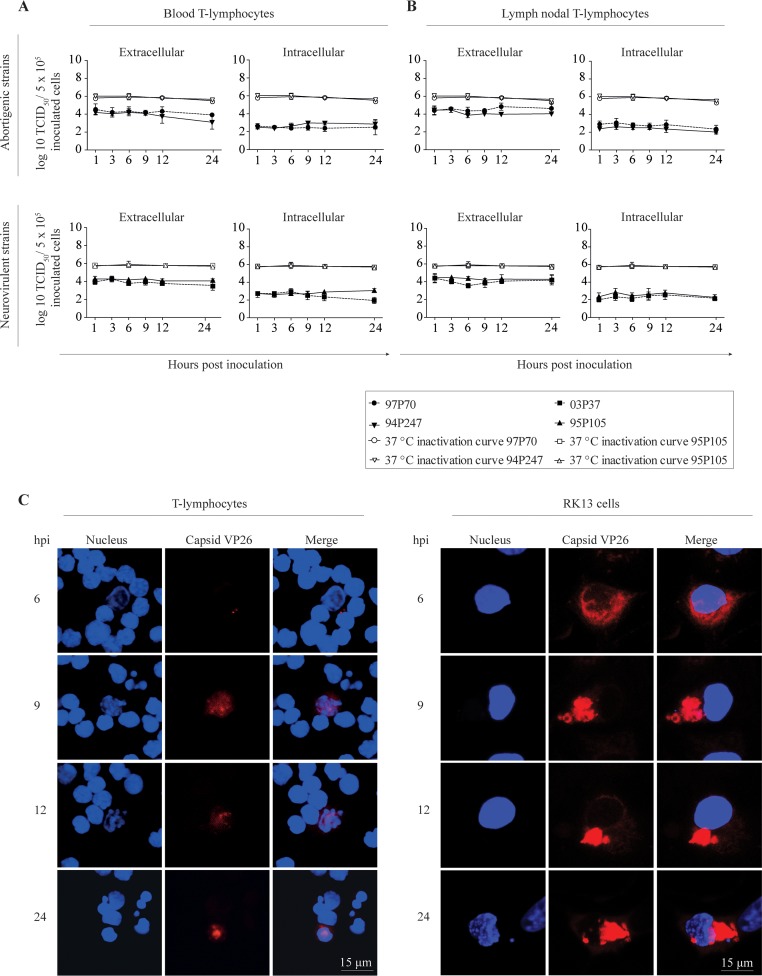
Kinetics of EHV1 production in blood (A) and lymph nodal (B) T lymphocytes. The upper panels show the abortigenic EHV1 variants (97P70 and 94P247), and the lower panels show the neurovirulent EHV1 variants (03P37 and 95P105). Data represent means plus SD for three independent experiments. (C) The spatiotemporal distribution of viral nucleocapsids in blood T lymphocytes is shown in red. Nuclei were counterstained blue (Hoechst dye).

**FIG 8:**
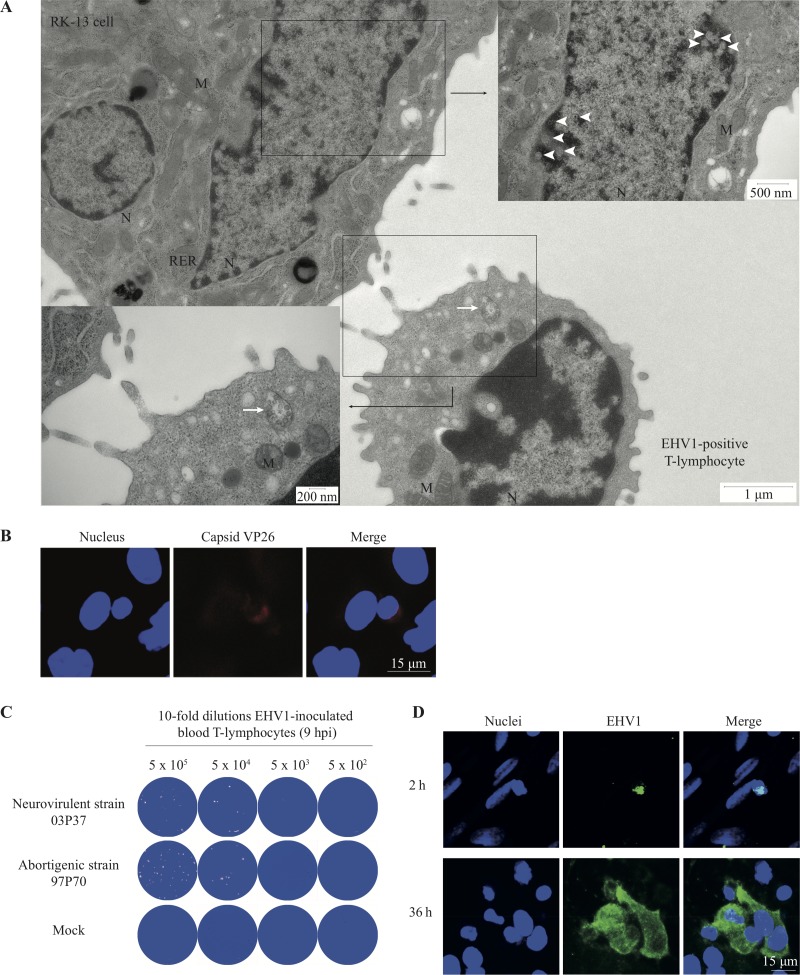
EHV1-infected T lymphocytes release nucleocapsids from the nucleus and transfer infection to an adjacent target cell. (A) Transmission electron micrographs of close cell-cell contacts between an EHV1-positive T lymphocyte and an RK13 cell. The arrow indicates the anterograde transport of EHV1 nucleocapsids budded into cellular vesicles toward the site of cell-cell contact. The arrowheads indicate viral nucleocapsids at the nucleus of the engaged RK13 cell. N, nucleus; M, mitochondria; RER, rough endoplasmic reticulum. (B) Contact between an EHV1-positive T lymphocyte and an RK13 cell activates the release of entrapped red-labeled nucleocapsids in the T cell nucleus. (C) Representative images of EHV1 plaques in crystal violet-stained RK13 cell monolayers after cocultivation with a 10-fold dilution of EHV1-inoculated T lymphocytes. (D) Representative confocal images of cocultivated EHV1-inoculated T lymphocytes and equine venous endothelial cells at 2 h and 36 h of cocultivation. EHV1 proteins are stained green. Nuclei are counterstained blue (Hoechst dye).

To determine whether EHV-1 replication in T cells is productive, we collected cells and supernatants at different time points postinoculation and performed virus titrations. No significant changes in extracellular and intracellular virus titers were observed in blood and lymph nodal T lymphocytes over time postinoculation (Fig. 7A and B). These results suggest a block in viral assembly and/or egress during EHV1 T-lymphocyte infection. To analyze this further, T lymphocytes were inoculated with the RacL11-EHV1 strain expressing a monomeric red fluorescent protein (mRFP1)-labeled VP26 protein (Fig. 7C). At 6 hpi, red fluorescent capsid proteins were detected in the cytoplasm of infected T lymphocytes. By 9 hpi, capsid proteins formed a diffuse intranuclear signal, which intensified into globular structures at 12 and 24 hpi (Fig. 7C, left panel). RK13 cells were used as a control cell line and demonstrated nuclear egress of the red-labeled capsid proteins (Fig. 7C, right panel). Consequently, we hypothesized that cell-cell contact might activate viral egress in EHV1-inoculated T lymphocytes in order to transmit infectious virus to the contact target cell. In the first assay, we cocultured EHV1-inoculated T lymphocytes at 9 hpi with RK13 cells and analyzed the coculture by transmission electron microscopy (TEM) imaging and immunofluorescence staining. Cocultures demonstrated the release of (red-labeled) nucleocapsids from the nucleus and the transfer of virions to RK13 cells within 2 h of cocultivation (Fig. 8A and B). With a viral plaque assay, we showed that approximately 0.3% of EHV1-positive blood T lymphocytes were productive at 9 hpi (Fig. 8C). To confirm the transfer of EHV1 from T lymphocytes to target cells, we incubated EHV1-inoculated T lymphocytes with venous endothelial cells (EC). Approximately 0.2% of the EHV1-positive T lymphocytes were able to transfer virus and productively infect EC (Fig. 8D). No significant differences in the transfer of EHV1 from T lymphocytes to target cells were observed between EHV1 strains (data not shown).

### Infection of T lymphocytes occurs more efficiently via cell-to-cell spread than by cell-free virus infection. (i) Viral transfer from infected RK13 cells to equine T lymphocytes.

**FIG 9:**
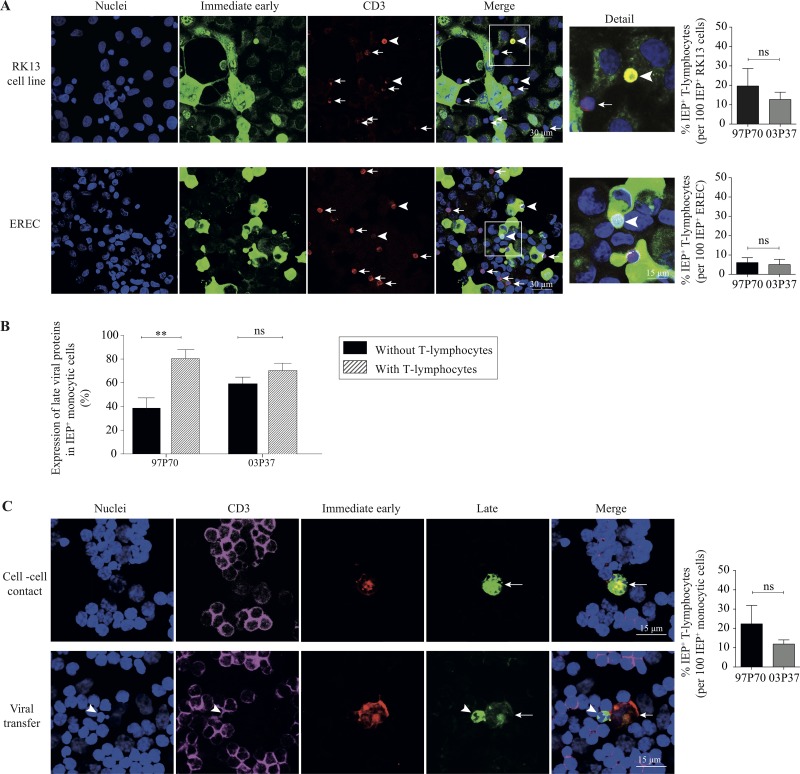
Cell-cell transfer facilitates efficient EHV1 infection of T lymphocytes. (A) RK13 cells (upper panels) and EREC (lower panels) were inoculated with the abortigenic (97P70) or neurovirulent (03P37) EHV1 variant. At 12 (RK13 cells) or 24 (EREC) hpi, cells were cocultured with primary T lymphocytes in the presence of neutralizing antibodies. After 2 h of cocultivation, RK13 cells, EREC, and adherent T cells were fixed with methanol. IEP and the CD3 T cell marker were visualized in green and red, respectively. Nuclei were counterstained blue (Hoechst dye). Arrows indicate adherent T lymphocytes, and arrowheads indicate EHV1-positive T cells. The right panels show the number of IEP-positive T lymphocytes per 100 EHV1-positive RK13 cells or EREC. (B) Expression of EHV1 late proteins in infected monocytic CD172a^+^ cells after 24 h of coculture with primary T lymphocytes. (C) Confocal images of cocultures of monocytic cells and T lymphocytes are shown in the left panels. Triple immunofluorescence staining of the CD3 T cell marker (purple), IEP (red), and late viral proteins (green) was performed to analyze the expression of late viral proteins upon cell-cell contact and the viral transfer between EHV1-infected monocytic cells and T lymphocytes. Nuclei were counterstained blue (Hoechst dye). The graph on the right represents the number of IEP-positive T lymphocytes per 100 EHV1-positive monocytic cells. All experiments were performed with blood-derived T lymphocytes from three different horses. Data represent means plus SD. **, *P* < 0.01; ns, no statistically significant differences.

The infection of leukocytes is a critical step in the viral dissemination in the host. However, the percentage of EHV1-positive T lymphocytes after inoculation with cell-free virus remained low and inefficient. Therefore, we hypothesized that T cells are more efficiently infected via direct cell-to-cell spread. RK13 cells were inoculated with EHV1 (MOI of 1). At 12 hpi, the cells were washed in citrate buffer and cocultured for 2 h with primary T lymphocytes (ratio of 1:1) in the presence of neutralizing antibodies. Nearly 90% of the RK13 cells were EHV1 positive. We found that approximately 15% (strain 03P37) and 20% (strain 97P70) of EHV1-positive RK13 cells were able to transmit virus to T lymphocytes. Representative confocal images and a graph are shown in Fig. 9A (upper panel).

### (ii) Viral transfer from infected primary respiratory epithelial cells to T lymphocytes.

Previous research demonstrated virus-induced recruitment of immune cells to the upper respiratory tract, the primary site of EHV1 replication (6, 7, 14). Here we analyzed whether EHV1-infected respiratory epithelial cells (EREC) can transfer virus to T lymphocytes in the presence of neutralizing antibodies. Therefore, EREC were inoculated with EHV1 at an MOI of 5. EREC were processed as described above. We found that 12.5% ± 8.3% and 11.7% ± 7.9% of EREC were infected with the abortigenic and neurovirulent strains, respectively. Representative confocal images are shown in the lower panel of Fig. 9A. The graph in the lower panel of Fig. 9A demonstrates that 100 EHV1-infected EREC were able to transfer virus to approximately 5 T lymphocytes within 2 h of coculture, independent of the strain used.

### (iii) Viral transfer from infected monocytic CD172a^+^ cells to equine T lymphocytes.

Next, we determined whether monocytic cells, known as the main target cells for EHV1, could transmit infectious virus to T lymphocytes. At 12 hpi, autologous monocytic cells were washed with citrate buffer and coincubated with the primary T lymphocytes at a ratio of 1:10 in the presence of neutralizing antibodies. Both cell types were collected after 24 h of cocultivation. T lymphocytes were distinguished from the monocytic cells by staining with a specific CD3 T cell marker. At 12 hpi, 3.9% ± 1.1% and 5.1% ± 1.6% of the monocytic cells inoculated with the abortigenic and neurovirulent strains, respectively, were IEP positive. This is consistent with previous results by Laval et al. (9, 15). Cell-cell contact between the monocytes and T cells increased viral replication in monocytic cells compared to that in the single-cultivated monocytes. This was demonstrated by increased expression of the late viral proteins, as shown in Fig. 9B. Indeed, we detected that 100 EHV1-positive monocytic cells transmitted infection to 22.3 ± 9.6 (abortigenic) and 11.9 ± 2.2 (neurovirulent) T lymphocytes within 24 h of coculture (Fig. 9C).

Taken together, our results demonstrate that cell-cell contacts facilitate the transmission of virus between EHV1-inoculated permissive RK13 cells, primary EREC, autologous monocytic cells, and T lymphocytes in the presence of protective immunity.

### Late viral proteins hijack the secretory pathway of T lymphocytes and orchestrate viral egress upon cell-cell contact.

**FIG 10:**
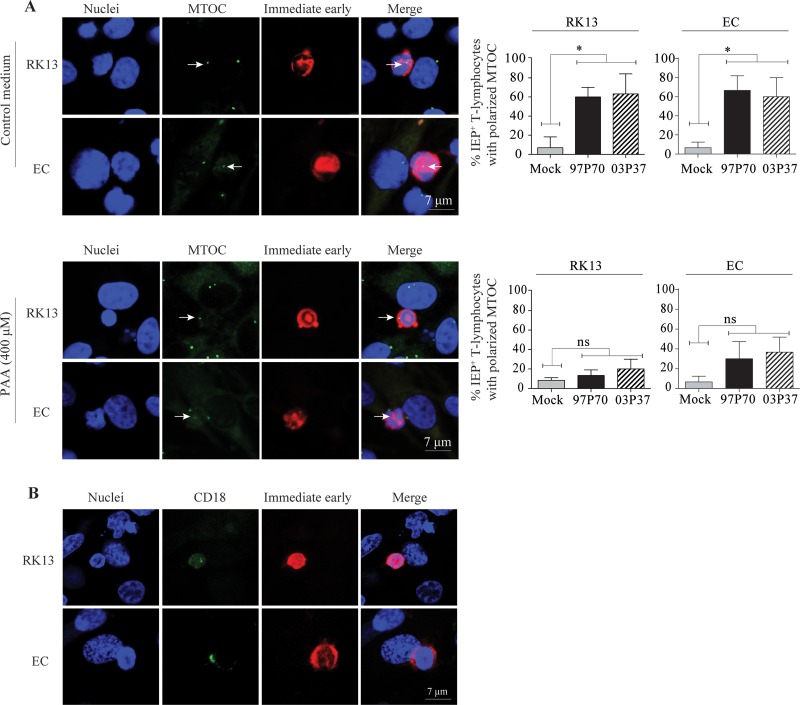
EHV1 misuses the regulated secretory pathway of T lymphocytes to promote its egress. (A) The graph in the upper panel shows enhanced MTOC polarization toward the site of contact with an RK13 cell or EC upon EHV1 infection. Blocking the transcription of late viral proteins with phosphonoacetic acid (PAA) significantly reduced the MTOC polarization (graph in lower panel). Representative confocal images are shown in the left panels. MTOC is stained green, and immediate early proteins (IEP) are stained red. Nuclei are counterstained blue (Hoechst dye). Arrows indicate the MTOC of the infected T lymphocytes. (B) Active LFA1 (CD18) is enriched at the site of contact between EHV1-infected T lymphocytes and RK13 cells or EC. LFA1 is stained green, and IEP is stained red. Nuclei are counterstained blue (Hoechst dye).

We demonstrated that direct cell-cell spread of EHV1 is an efficient mode of dissemination to and from T lymphocytes in the presence of neutralizing antibodies. However, the mechanisms by which EHV1 proteins are directed toward intercellular contacts remain unclear. Many viruses, including human immunodeficiency virus (HIV) and human T-lymphotropic virus (HTLV), show a directed egress at the cell-cell contact site, which is coordinated by the regulated secretory pathway (41–44). We therefore hypothesized that EHV1 might exploit elements of this pathway to orchestrate egress from T cells and promote cell-to-cell spread. A noticeable feature of the regulated secretion of T lymphocytes at sites of cell-cell contact is the polarization of the microtubule organizing center (MTOC) toward the target cell (41, 45–47). To investigate such MTOC polarization, we incubated EHV1-inoculated primary T lymphocytes (9 hpi) with RK13 cells or EC for 2 h. We examined T cell polarization toward the sites of cell-cell contact by immunofluorescence staining of MTOC and IEP, followed by confocal microscopy. Confocal images of MTOC reorientation toward the cell-cell junction in EHV1- or mock-inoculated T lymphocytes are shown in Fig. 10A, upper panel. Upon T lymphocyte-RK13 cell contact, 60% ± 10% and 63% ± 21% of the MTOC in abortigenic and neurovirulent EHV1-positive T lymphocytes aligned to the site of cell-cell interaction, compared to 7% ± 12% in EHV1-negative T lymphocytes (*P* < 0.05). Similarly, alignment of the MTOC in EHV1-positive T cells to the site of cell contact with the venous endothelial cells occurred in 67% ± 15% and 60% ± 20% of the cells upon infection with the abortigenic and neurovirulent strains, respectively. This in contrast to 7% ± 6% of EHV1-negative T cells with a polarized MTOC. By blocking viral DNA replication with phosphonoacetic acid (PAA), we significantly reduced the reorientation of the MTOC toward the RK13 cells and EC, to 13% ± 6% (*P* < 0.05) and 30% ± 17% (*P* < 0.05), respectively, in T lymphocytes inoculated with the abortigenic strain and 20% ± 10% (*P* = 0.05) and 37% ± 15% (*P* < 0.05), respectively, for the neurovirulent EHV1 strain (Fig. 10A, lower panel). This T cell reorientation suggested viral transmission at a structured area of cell-cell contact, termed the viral synapse (VS). Previous studies demonstrated the central role of the active form of LFA1 (CD18) on the lymphocyte surface in promoting synapse formation (48, 49). Here we showed the role of active LFA1 in the interaction of EHV1-infected T cells with adjacent target cells by immunofluorescence staining. Figure 10B shows the enrichment of activated LFA1 at the site of contact between the infected T lymphocyte and its target RK13 cells or EC.

**FIG 11:**
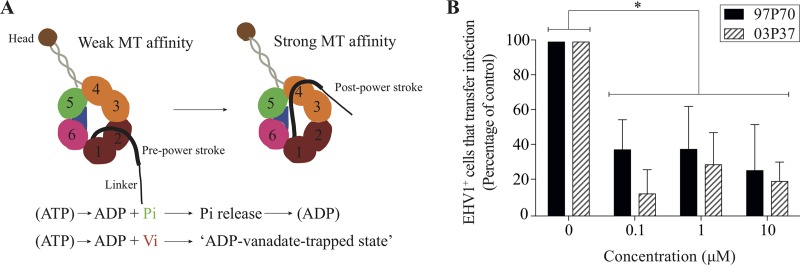
Viral antigens hijack the dynein motors to transport viral progeny to the viral synapse. (A) Effects of vanadate (V_i_) on the activity of the dynein motor. Hexameric rings linked into one large polypeptide form the core of the dynein motor. The transitions between the pre- and post-power-stroke states are dependent on the highly coordinated interactions between the structural components of the dynein head. Upon ATP binding, the dynein head releases the microtubule (MT) and accelerates hydrolysis of ATP to ADP + P_i_. This results in the conformational changes of the linker into the pre-power stroke. Once P_i_ is released, the stalk resumes its high-affinity conformation that locks the dynein head onto the MT. The linker swings from its pre-power-stroke position (AAA2) to the post-power-stroke position (AAA4) when ADP is released. V_i_ replaces P_i_ in the dynein-ADP-P_i_ complex, resulting in a covalent ADP entrapment and a dead-end kinetic block of the ATPase of the dynein motor. (B) At 9 hpi, EHV1-inoculated T cells were washed with citrate buffer, followed by 1 h of treatment with 0, 0.1, 1, or 10 μM V_i_ prior to cocultivation with RK13 cells. After 48 h of cocultivation, the plaques were fixed and stained with crystal violet. For each condition, viral plaques were counted and graphed as percentages of the control level. Data represent means plus SD for three independent experiments. *, *P* < 0.05.

**FIG 12:**
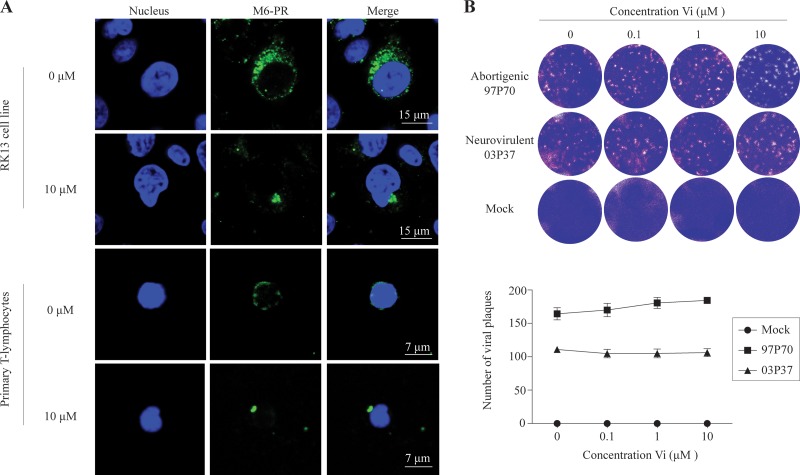
Inorganic vanadate (V_i_) disrupts the cellular dynein motors but does not interfere with viral replication. (A) Expression of mannose 6-phosphate receptor (M6-PR) in mock- or V_i_-treated RK13 cells or equine T lymphocytes. For mock-treated cells, a scattered expression pattern of M6-PR (green) was observed. Upon treatment with 10 μM V_i_, M6-PR clustered in the juxtanuclear region. Nuclei were counterstained blue (Hoechst dye). (B) (Top) A viral plaque assay with RK13 cells, cell-free virus, and different concentrations of V_i_ was carried out, and plaques were visualized by crystal violet staining. (Bottom) Numbers of viral plaques. Data represent means ± SD for three independent experiments performed in triplicate.

Following the tubulin cytoskeleton polarization, we hypothesized that viral antigens hijack dynein and/or kinesin motors to transport progeny virus toward the VS. To test this, EHV1-inoculated T lymphocytes were pretreated with increasing concentrations (0, 0.1, 1, and 10 μM) of a dynein motor inhibitor, inorganic vanadate (V_i_), at 9 hpi, prior to cocultivation with RK13 cells. Figure 11A shows the mechanism of action of V_i_. V_i_ is a transition state analog in phosphoryl transfer actions, as it forms a structure similar to that of phosphate ester (P_i_) during hydrolysis (ADP-V_i_) (50, 51). Subsequently, dynein motors are biochemically trapped in their ATPase cycles (52). To verify the V_i_-mediated disruption of the dynein motors, we visualized the expression of mannose 6-phosphate receptor (M6-PR) on the cell surface (Fig. 12A). To exclude direct effects of V_i_ on viral replication, a viral plaque assay based on cell-free virus and RK13 cells was performed (Fig. 12B). The results for the V_i_-treated EHV1-inoculated T cells are shown as percentages of the control level in Fig. 11B. Both the abortigenic and neurovirulent EHV1 strains showed a significant reduction in the percentage of EHV1-infected T lymphocytes able to transfer infection to RK13 cells when the V_i_ concentration increased from 0 μM to 10 μM (*P* < 0.05).

Taking all these observations together, we may conclude that upon cell-cell contact a late viral protein(s) orchestrates the transfer of infectious virus from T lymphocytes to target cells by stimulating MTOC polarization, accumulation of active LFA1, and thus formation of a viral synapse (VS) between the two cell types. Once the VS is formed, EHV1 hijacks the microtubule network in a dynein-dependent manner to accelerate anterograde transport of new progeny virus toward the plasma membrane.

## DISCUSSION

**FIG 13:**
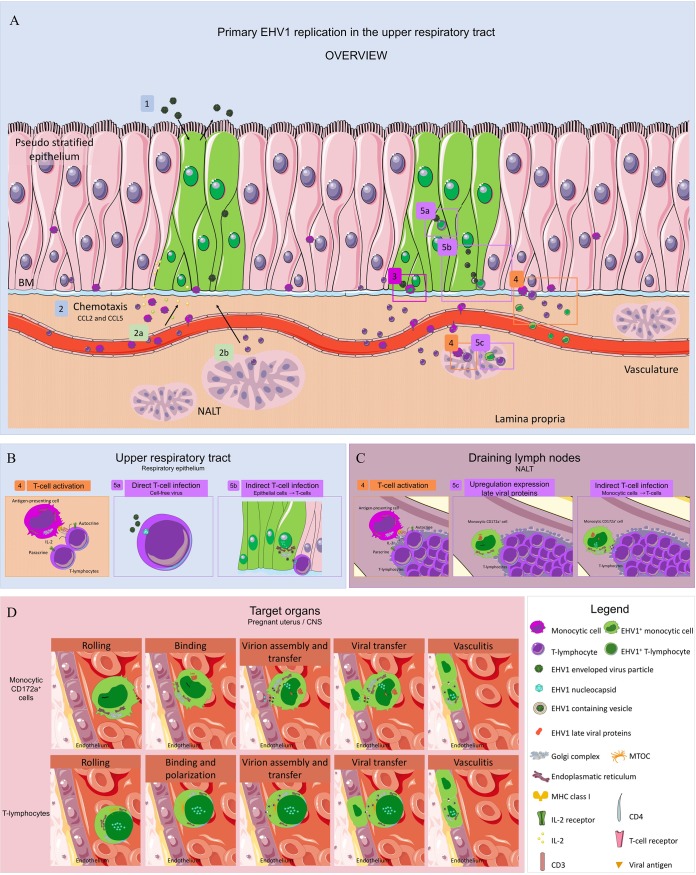
Hypothetical model of EHV1 infection of T lymphocytes. (A) (1) During primary EHV1 replication in the URT, (2) monocytic cells and T lymphocytes are attracted to the site of infection. (3) Extravasated monocytic cells become infected by cell-free virus at the URT. (B and C) (4) Patrolling monocytes and dendritic cells present viral antigen to T lymphocytes at the level of the URT or draining lymph nodes, resulting in T cell activation (5). Activated T lymphocytes become efficiently infected either directly by cell-free virus (5a) or indirectly by cell-to-cell transfer (5b and 5c). (D) Once infected, monocytic cells and T lymphocytes migrate to the target organs. Upon binding to the target endothelium, viral replication in monocytic cells is again activated. Progeny virus is assembled and transferred to the endothelium. On binding of infected T lymphocytes to target endothelium, a late viral protein(s) orchestrates T cell polarization, viral assembly, and transfer to the adjacent endothelium. Infection of the endothelium ultimately results in vasculitis.

During EHV1 infection, extravasated monocytic cells and T lymphocytes are attracted to the infected respiratory mucosa within 24 to 36 h after infection (Fig. 13A) (6, 7, 14). Numerous reports have demonstrated that lymphocytes are important in clearing herpesvirus infections, as CD8^+^ T lymphocytes play a prominent role in the elimination of viruses. Infecting the cellular arm of the immune response is therefore an effective and refined strategy for evading the immune responses to promote viral dissemination in the host, evade host clearance, and establish latency. Thus, it is not surprising that several alphaherpesviruses, such as HSV and VZV, have developed T-lymphocyte tropism as one of multiple immune evasion mechanisms (30, 53). For instance, by infecting T cells, HSV modulates TCR signaling, which prevents T cell cytotoxicity and mediates the cytokine production toward an immunosuppressive phenotype (54). Alternatively, T cell infection by VZV is critical for the virus to disseminate through the host during viremia, ultimately resulting in cutaneous vesicular rash (55).

Monocytic CD172a^+^ cells have been shown to be the main carrier cell of EHV1 during primary viral infection (6, 8, 56). EHV1 misuses CD172a^+^ cells as a “Trojan horse” to migrate in the host to reach the target organs, accordingly evading the host immune response (9). Alongside monocytic cells, T lymphocytes are described as potential EHV1 target cells (8, 22, 23, 57). However, until present, little information was available about the T cell tropism of EHV1. A previous *in vitro* study from van der Meulen et al. (8) demonstrated that mitogen-activated T lymphocytes isolated from the blood were more susceptible than resting T cells to the abortigenic 97P70 EHV1 strain. Mitogen-induced activation of specific cell cycle events was found to result in enhanced viral replication. In addition, mitogens triggered the formation of close intercellular contacts, further facilitating viral cell-to-cell transmission. However, the questions of whether both abortigenic and neurovirulent EHV1 variants acquired the ability to infect T cells and which T cell population (CD4^+^ and/or CD8^+^) is more susceptible remained unanswered. Moreover, whether and how T lymphocytes transfer infection to the endothelium of the target organs were still unclear. In the present study, we provided evidence that blood- and lymph node-derived T lymphocytes are susceptible to EHV1 infection *in vitro*. We demonstrated that blood and lymph nodal T lymphocytes were susceptible to abortigenic and neurovirulent EHV1 strains. Moreover, we found that blood-derived T cells were more susceptible to EHV1 infection than lymph nodal T lymphocytes. One plausible explanation is the different cell surface phenotypes of T lymphocytes derived from lymph nodes and the peripheral blood, which might be used by EHV1 to bind and/or enter T lymphocytes (58). Indeed, T lymphocytes from peripheral organs contain more T cells with the effector/memory phenotype than the blood-derived T cells (59). The observations of phenotypic differences between the abortigenic and neurovirulent variants are in line with a previous study from Vandekerckhove et al. (6). They showed that abortigenic virus-infected immune cells were equally identified as monocytic CD172a^+^ cells and T lymphocytes. In contrast, the vast majority of neurovirulent EHV1-infected immune cells belonged to the CD172a^+^ cell lineage, followed by a smaller portion of infected T cells. These results suggest different immune evasive strategies among the circulating EHV1 phenotypes.

Next, we demonstrated that CD4^+^ cells are more susceptible to EHV1 infection than CD8^+^ cells. These results are in accordance with previous reports for other alphaherpesviruses and are consistent with earlier EHV1 studies (6, 33, 60, 61). Moreover, CD4 has been demonstrated to be a critical component of the viral receptor for certain betaherpesviruses, including human herpesvirus 7 (62, 63). This leads to the promising role of CD4 as a (co)receptor for EHV1. To address this possibility, more research is needed. Using CD4 as a (co)receptor would not be a surprising mechanism of EHV1, since 64% of circulating T lymphocytes in the adult horse express CD4, and only 18% are CD8^+^ (64). Moreover, previous research demonstrated that CD4^+^ Th_1_ cells predominantly infiltrate at the mucosal surfaces and clear HSV infection (65, 66). To overcome viral elimination, many herpesviruses, including EHV1, have evolved a CD4^+^ T cell tropism.

We also demonstrated that IL-2 stimulation affects the susceptibility of T lymphocytes to EHV1 and increases the number of EHV1-positive T cells. Upon a mucosal viral infection *in vivo*, recruited inflammatory immune cells secrete IL-2 in the inflamed tissue. IL-2 promotes T cell proliferation and exerts effects on the cellular metabolism and glycolysis that are necessary for long-term T cell survival (67, 68). Many viruses, including EHV1, misuse T cell activation. First, we hypothesized that the IL-2 receptor signaling pathway upregulates a putative EHV1 receptor on the T cell surface. Current research to address this is ongoing. Second, it is possible that the activation of T lymphocytes enhances downstream signaling pathways, which the virus misuses to mediate its intracellular transport, including the nuclear translocation of immediate early proteins. This induces the upregulation of early and late viral protein expression, resulting in enhanced viral replication. A third theory is that IL-2-induced transcription factors transactivate the transcription of multiple viral genes. Visual tracking of fluorescently labeled viral proteins and molecular analyses of viral gene expression upon IL-2 stimulation would be of great interest for future research.

We found that EHV1-infected T lymphocytes are going into apoptosis starting from 9 hpi. Many cells will undergo programmed cell death following viral infection, to abort the production and release of viral progeny. These results suggest that the distinct mechanisms for modulation of host cell apoptosis evolved by EHV1 are not sufficient to prevent this biological event. Since the programmed cell death of infected T cells leads to the release of apoptotic bodies, we cannot rule out the prevalence of large apoptotic bodies in our inoculated T cell population. Apoptosis of T lymphocytes may account for lymphopenia and immunosuppression following EHV1 viremia *in vivo* from days 2 to 7 (69). Based on the small fraction of infected T lymphocytes that remain alive at 24 hpi, we hypothesized that memory T cells are critical for maintaining the infection in the T cell population and carrying the virus to the target organs. However, indirect immunofluorescence staining of the general memory T cell marker CD45RO and of viral proteins did not show any colocalization (data not shown). Therefore, which CD4^+^ T cell subset preserves EHV1 infection still needs to be defined.

Laval et al. (9) demonstrated that the replication of abortigenic EHV1 is silenced in monocytic CD172a^+^ cells, as the expression of late viral proteins is hampered in these cells (Fig. 13D). In the present study, we highlighted new and distinct immune evasion strategies of EHV1 in T lymphocytes. Upon T cell infection, IE and late (gB and gC) viral proteins were consistently detected at 1, 3, and 6 hpi, respectively, including membrane expression of viral glycoproteins, suggesting the occurrence of efficient viral replication. During infection, newly synthesized viral proteins are transported to and incorporated into different cellular lipid bilayers, including the plasma membrane (70). These membrane-bound viral proteins render the recognition of infected cells by antibodies, resulting in cell- and complement-mediated lysis of the infected cells (71). Interestingly, viral glycoproteins cluster at the plasma membrane, forming aggregates on one side of the cell. These results indicate sequestration of the membrane-bound viral proteins, leading to infected T lymphocytes without visually detectable levels of viral glycoproteins on the plasma membrane. These results are in line with previous studies of pseudorabies virus (PrV)-infected monocytic cells (70, 72). Given the highly immuno-evasive character of herpesviruses, this viral protein sequestration might be interpreted as an immuno-evasive strategy.

Despite the efficient viral replication in T lymphocytes, we observed a restriction in the production of viral progeny, resulting in a nonproductive infection in lymphocytes. By imaging EHV1 encoding VP26 tagged with mRFP, we visualized the accumulation of viral capsids in the nucleus. Our results are in line with a study by Reichelt et al. (73), who showed the nuclear entrapment of newly assembled viral capsids of VZV, a virus closely related to EHV1, in neuronal cells. Nuclear accumulation and inhibition of progeny virus release might be interpreted as an intrinsic antiviral defense. Alternatively, it might benefit the virus to persist and disseminate unnoticed in the host. Indeed, we showed that once an infected T cell binds to a secondary target cell, such as an endothelial cell of the pregnant uterus or the CNS, late viral protein expression induces the reorientation of the MTOC toward the contact cell and the formation of a virological synapse (VS). This mechanism allows EHV1 to hijack dynein motors to facilitate the anterograde transport of newly assembled virus progeny toward the VS and subsequent infection of the target cell (Fig. 13D). Here we demonstrated the involvement of the cellular protein LFA1 in the formation of a VS. Since Spiesschaert et al. (74) reported the importance of gB in the viral transfer between peripheral blood mononuclear cells (PBMC) and EC, we assume that viral proteins play a pivotal role in the formation or maintenance of the VS. More research is needed to verify which viral proteins might be involved.

Egress of new virions from the nucleus comprises a complex process that involves many viral and cellular participants, such as kinases. In HSV-1-infected cells, pUL34, pUL31, and pUS3 play a pivotal role in nuclear egress. The pUL34 and pUL31 proteins form a complex phosphorylated by pUS3, mediating the rearrangement of the nuclear lamina (75). Apart from the indirect role of pUS3, pUS3 and the host protein kinase C (PKC) independently induce direct defects in the nuclear lamina, stimulating nuclear egress (76, 77). Since pUS3 is conserved among all alphaherpesviruses, it is not unexpected that pUS3 of EHV1 has been associated with nuclear egress (78). In this study, we hypothesized that contact of an EHV1-infected T cell with a target cell signals the activation of nuclear PKC and viral pUS3, resulting in the recruitment of viral capsids to the nuclear membrane and reorganization of the nuclear lamina, followed by T cell polarization and the nuclear egress of EHV1 capsids. Hijacking T cell polarization and the dynein machinery permits lymphotropic viruses, such as HIV and HTLV, to acquire temporal and spatial control over virus assembly and egress (41, 43, 45–47, 79). Interestingly, we showed that both EHV1 phenotypes use a similar strategy in T lymphocytes. The fact that many lymphotropic viruses, including herpesviruses, bridle the regulated secretory pathway of lymphocytes suggests an ancient mechanism for remaining in the host (41, 80). In our understanding, counteracting the nuclear release of EHV1 capsids in T lymphocytes would be a valuable therapeutic tool for reducing viral infection of the target endothelium.

Viral spread via cell-cell contacts is a common strategy used by herpesviruses to remain undetected by the immune system. This immune evasion strategy is important not only for viral transmission from infected PBMC to EC. Here we found that EHV1 can also efficiently infect T cells via direct transfer from infected respiratory epithelial cells and CD172a^+^ monocytic cells (Fig. 13B and C). The contact between an infected monocytic cell and a T lymphocyte enhances viral replication, resulting in an efficient viral transfer to the engaged T lymphocytes. This strategy provides a transient advantage to the virus, allowing it to successfully cross the basement membrane and (in)directly enter the circulation via the draining lymph nodes. Viral transfer between monocytes and T lymphocytes may increase the quantity of circulating infected and nonproductive immune cells to maximize viral spreading to reach the endothelium of the target organs.

In conclusion, our data demonstrate that T lymphocytes are important target cells for both abortigenic and neurovirulent EHV1 variants. EHV1 uses a strategy for infecting T cells that is completely different from that for infecting monocytic cells. In support of the Red Queen hypothesis, which indicates that coevolving species need to evolve constantly to keep up with each other, we propose that abortigenic EHV1 variants have coevolved longer with their host than neurovirulent variants, which is in line with the evolutionary theory of Nugent et al. (13). This long coevolution resulted in a more advanced adaptation to the host, resulting in additional, sophisticated immune evasion strategies, such as silencing of viral replication in monocytic cells. In contrast, infecting T lymphocytes appears to be an immune evasion mechanism that is universally exploited by many viruses. We showed that progeny viral capsids accumulate in the nuclei of T lymphocytes. We suggest that cellular contact signals the late viral proteins to discontinue nuclear entrapment and to facilitate viral envelopment, egress, and transfer to the engaged target cell. Understanding the evolutionary combat between EHV1 and the immune surveillance of the horse is of great interest for the development of new therapeutics to prevent viral dissemination in the horse.

## MATERIALS AND METHODS

### Viruses.

The EHV1 abortigenic strains 94P247 and 97P70 were originally isolated in 1994 and 1997, from the lungs of aborted fetuses (81, 82). Both strains encode an asparagine at amino acid position 752 (N_752_) (13). The neurovirulent 95P105 and 03P37 EHV1 strains were first isolated in 1995 and 2003, from the blood of paralytic horses (81, 83). Both strains encode an aspartate at amino acid position 752 (D_752_) (13).The monomeric red fluorescent protein (mRFP1)-labeled RacL11 strain was constructed by inserting mRFP1 into the VP26 capsid protein of the RacL11 strain and was kindly provided by N. Osterrieder (Germany) (84). The RacL11 EHV1 strain was originally obtained from an aborted foal (85).

### Donor horses and cells. (i) Isolation of equine blood CD172a^+^ cells and T lymphocytes.

Peripheral blood was sampled from the external vena jugularis into heparin (Leo, Zaventem, Belgium) at a final concentration of 15 U ml^−1^. The Ethical Committee of the Faculty of Veterinary Medicine, Ghent University (application EC2017/118), approved the collection of blood. Fresh blood was diluted in an equal volume of Dulbecco’s phosphate-buffered saline (DPBS) without calcium and magnesium (Gibco, Invitrogen, Paisley, United Kingdom). Peripheral blood mononuclear cells (PBMC) were isolated by density centrifugation on Ficoll-Paque (*d* = 1.077 g ml^−1^) (GE Healthcare, Life Sciences) at 800 × *g* for 30 min at 18°C. The interphase band, containing the PBMC, was collected and washed three times with DPBS. Cells were resuspended in complete medium, which was based on RPMI medium (Gibco) and supplemented with 5% newborn fetal calf serum (FCS) (Gibco), 1% nonessential amino acids, 1% sodium pyruvate, 1% penicillin-streptomycin, and 0.5% gentamicin (Gibco). Afterwards, PBMC were seeded at a concentration of 5 × 10^6^ cells per ml in a plastic petri dish and cultivated at 37°C with 5% CO_2_. After 2 h, nonadhering leukocytes were removed by washing cells three times with RPMI medium, and the adherent cells were cultured in complete medium. Nonadherent cells were washed 3 times with ice-cold RPMI medium. The cell pellet was incubated with 400 μl of mouse anti-horse pan-B-cell (IgG1; 1:50) (clone CVS36; Bio-Rad, USA), anti-horse CD4 (IgG1; 1:50), or anti-horse CD8α (IgG3; 1:50) monoclonal antibodies (MAbs) (Monoclonal Antibody Center, USA), diluted in ice-cold DPBS, for 1 h at 4°C with gentle agitation. Afterwards, cells were washed once in magnetic-activated cell sorting (MACS) buffer, containing DPBS supplemented with 1 mM EDTA and 5% newborn FCS, and then incubated with 400 μl rat anti-mouse IgG microbeads (MACS Miltenyi Biotec, Bergisch Gladbach, Germany) diluted in MACS buffer (1:5) for 1 h at 4°C with gentle agitation. Next, the cells were washed once in MACS buffer and resuspended in 3 ml MACS buffer for application to the MACS column. The unbound cells of the pan-B-cell antibody-incubated T lymphocytes were collected from the column and consisted of >90% CD3^+^ cells as assessed by flow cytometry (data not shown) after incubation with an anti-CD3 MAb (IgG1; 1:50) (clone UC-F6G; UC Davis, Davis, CA, USA) directed against cells from the T cell lineage followed by goat anti-mouse IgG conjugated to fluorescein isothiocyanate (FITC) (1:200) (Molecular Probes). The positively selected T lymphocytes for CD4 and CD8 consisted of >90% CD4^+^ and CD8^+^ cells as analyzed by flow cytometry (data not shown). All T lymphocytes were cultured in complete medium supplemented with 4 U ml^−1^ human recombinant interleukin-2 (hIL-2) (R&D Systems) and 50 mM β-mercaptoethanol (β-ME) (Gibco) unless specified differently.

### (ii) Isolation of equine lymph nodal T lymphocytes.

Pulmonary lymph nodes were collected from three horses at the slaughterhouse and cooled transported to the lab in DPBS supplemented with 1% gentamicin, 1% penicillin-streptomycin (Gibco), 1% kanamycin (Sigma-Aldrich, St. Louis, MO), and 0.5% amphotericin B (Bristol-Myers Squibb), as described previously (86). The isolation of the lymph nodal PBMC was adapted from the protocol described by Matheu and Cahalan (87). Redundant fat and connective tissue were removed from the lymph nodes. The lymph nodes were transferred to a new petri dish placed on ice containing RPMI medium. Tissues were cut into small pieces, transferred to a 70-μm cell strainer, and squeezed with the pestle of a 5-ml syringe until only waste tissue remained. Medium containing the cells was collected and transferred into a new 70-μm cell strainer to remove all cell clots. The cells were rinsed twice and layered onto Ficoll-Paque (*d* = 1.077 g ml^−1^) (GE Healthcare). The interphase band was collected, washed, and resuspended in 2 ml warm RPMI medium. Cells (1.5 × 10^8^) were subjected to nylon wool purification by loading onto a column containing scrubbed nylon fibers (Polysciences, Inc.), executed as described in the manufacturer’s guidelines. Briefly, the columns were equilibrated by washing with 20 ml RPMI medium with 10% newborn FCS, sealed, and incubated for 1 h at 37°C and 5% CO_2_. Washing medium was drained, and cells were loaded onto the column. The column was sealed and incubated at 37°C and 5% CO_2_ for 1 h. The nonadherent T lymphocytes were eluted with 10 ml warm RPMI medium, washed, and then separated by MACS as described previously. The purity of the CD3^+^ cell fraction was >90% (data not shown) as assessed by flow cytometry, as described above. Lymph nodal T lymphocytes were cultured in complete medium supplemented with 4 U ml^−1^ hIL-2 and 50 mM β-ME as described above.

### (iii) Equine venous endothelial cell culture.

Laval et al. (88) previously reported the isolation and immortalization of equine venous endothelial cells (EC). EC were grown to confluence in 8-well Lab-Tek II chamber slides (Thermo Scientific, Rochester, NY) coated with 10 μg ml^−1^ fibronectin from bovine plasma (Sigma-Aldrich). EC were cocultured with EHV1-inoculated T lymphocytes (1:1 ratio) to visualize, detect, and quantify T cell polarization, accumulation of adhesion molecules, and viral transfer toward the EC.

### (iv) Equine primary respiratory epithelial cells.

The method for isolation and cultivation of equine respiratory epithelial cells (EREC) was adapted from the protocol described by Quintana et al. (89). Briefly, equine tracheal tissues were washed twice with cold DPBS to remove red blood cells. Epithelial cells were isolated by an enzymatic digestion using gentle agitation in calcium- and magnesium-free minimal essential medium (MEM; Gibco) containing 1.4% pronase (Roche Applied Science, Indianapolis, IN) and 0.1% DNase I (Sigma-Aldrich). Tissues were incubated with the enzyme mix for 48 h. Cells were cultured in a plastic uncoated petri dish for 6 h in Dulbecco’s modified Eagle’s medium (DMEM)/F-12 medium (Gibco) containing calcium- and magnesium-free MEM, 1% penicillin-streptomycin, and 2.4 μg ml^−1^ insulin (Sigma-Aldrich) to reduce fibroblast contamination. EREC were stored in liquid nitrogen at a density of 2 × 10^6^ cells per cryovial until further use. For culture, we seeded the EREC into type IV collagen (Sigma-Aldrich)-coated 6-well plates (Gibco) in DMEM/F-12 containing 5% non-heat-inactivated fetal bovine serum (Gibco), 1% calcium- and magnesium-free MEM, 1% penicillin-streptomycin, and 0.5% amphotericin B. After 24 h of culture, we removed the medium and cultivated the cells in epithelial cell medium containing DMEM/F-12 supplemented with 2% Ultroser G (Pall Life Sciences, Pall Corp., Cergy, France), 1% penicillin-streptomycin, and 0.5% amphotericin B. EREC were incubated to confluence in a humidified incubator at 37°C and 5% CO_2_.

### (v) Rabbit kidney epithelial (RK13) cells.

RK13 cells were purchased from the American Type Culture Collection (ATCC, Manassas, VA, USA) and were used in this study to examine viral transfer to and from primary T lymphocytes, to investigate T cell polarization and accumulation of adhesion molecules, and to analyze EHV1 replication by quantifying extracellular and intracellular virus titers at different time points postinoculation. Cells were maintained in MEM supplemented with antibiotics and 5% FCS.

### (vi) Cell viability.

Cell viability was determined by flow cytometry using 1 μg ml^−1^ propidium iodide (Sigma-Aldrich) prior to virus inoculation and was >90% in all cell populations. An *in situ* cell death detection kit (fluorescein) based on terminal deoxynucleotidyltransferase-mediated dUTP-biotin nick end labeling (TUNEL) was obtained from Roche (Mannheim, Germany) and used to detect DNA fragmentation induced by apoptotic signaling cascades in EHV1-positive T lymphocytes. The colocalization of viral protein expression and incorporated dUTP in T lymphocytes over time was analyzed by fluorescence microscopy (Leica DM RBE microscope; Leica Microsystems GmbH, Heidelberg, Germany). The number of TUNEL- and EHV1-positive cells was evaluated in five randomly chosen fields of 100 cells.

### EHV1 inoculation. (i) Infection of RK13 cells with EHV1.

RK13 cells were used as a control cell line and were inoculated with the mRFP1-VP26 RacL11 EHV1 strain at a multiplicity of infection (MOI) of 0.1 in 200 μl medium for 1 h at 37°C with 5% CO_2_. RK13 cells were rinsed twice with MEM and further cultured in MEM supplemented with 5% FCS and antibiotics. At 6, 9, 12, and 24 hpi, RK13 cells were fixed with 100% methanol for 20 min at −20°C.

### (ii) Infection of T lymphocytes with cell-free viruses.

Cell populations were inoculated with the 97P70, 94P247, 03P37, 95P105, or mRFP1-VP26 RacL11 EHV1 strain at an MOI of 5 in 400 μl complete medium for 1 h at 37°C with 5% CO_2_. T lymphocytes were gently washed twice with ice-cold RPMI medium and further cultured in complete medium. Mock infections were carried out in parallel.

### (iii) Infection of T lymphocytes through cell-cell spread.

***(a) EHV1-infected RK13 cells.*** Infection of T lymphocytes through cell-cell spread was assessed by inoculating monolayers of RK13 cells, cultivated on inserts, with the 97P70 or 03P37 EHV1 strain in 24-well plates at an MOI of 1 for 1 h at 37°C. The virus inoculum was removed, and cells were rinsed three times with MEM and incubated at 37°C for 12 h with 5% CO_2_. At 10 hpi, RK13 cells were washed with citrate buffer containing 40 mM citric acid, 135 mM NaCl, and 10 mM KCl (pH 3) for 1 min at room temperature (RT). Cells were washed three times with MEM to remove redundant citrate buffer. Complete medium supplemented with 4 U ml^−1^ hIL-2, 50 mM β-ME, and 5% hyperimmune horse serum (HHS) (derived from an EHV1-vaccinated horse; seroneutralization titer, 768), containing T lymphocytes, was added (ratio of 1:1). Cells were incubated for 2 h at 37°C and 5% CO_2_. RK13 cells and adherent T lymphocytes were directly fixed with 100% methanol (20 min, −20°C) at the end of the cocultivation.

**(b) EREC.** EREC were cultured in a 6-well plate. Confluent EREC were exposed to 2 ml EHV1 abortigenic strain 97P70 or neurovirulent strain 03P37 (MOI of 5) for 1 h at 37°C and 5% CO_2_. Virus particles were removed by washing the EREC three times with DMEM. Fresh EREC medium was added to the wells, and cells were incubated further. At 24 hpi, cells were washed with citrate buffer for 1 min at RT, followed by washing three times with DMEM. Complete medium supplemented with 5% HHS containing T lymphocytes was added at the apical surface for 2 h at 37°C and 5% CO_2_ (ratio of 1:1). Nonadherent T lymphocytes were collected, and EREC with possible bound T lymphocytes were detached from the plate by use of Accumax (Sigma-Aldrich) for 30 min at 37°C. All cells were fixed with 1% paraformaldehyde (PFA) for 10 min at RT. EREC and T lymphocytes were cytospin centrifuged and stored at 4°C until further processing.

**(c) EHV1-infected CD172a^+^ monocytic cells.** Monocytic cells cultured on poly-d-lysine hydrobromide (50 μg ml^−1^; Sigma-Aldrich)-coated inserts in wells of a 24-well tissue culture plate at a concentration of 5 × 10^5^ cells per ml were inoculated with the 97P70 and 03P37 EHV1 strains at an MOI of 5 in 200 μl complete medium for 1 h at 37°C and 5% CO_2_. Monocytic cells were gently washed twice with ice-cold RPMI medium and further cultured in complete medium. Mock inoculations were carried out in parallel. At 12 hpi, monocytic cells were washed with citrate buffer for 1 min at RT to neutralize cell-free virus particles. Cells were gently washed three times with RPMI medium. The EHV1-inoculated monocytic cells were incubated with autologous T lymphocytes at a ratio of 1:10 in complete medium supplemented with 4 U ml^−1^ hIL-2, 50 mM β-ME, and 5% HHS. Plates containing monocytic cells and T lymphocytes were incubated for 24 h on a rocking platform at 10 rpm. Monocytes and T lymphocytes were fixed with 1% PFA for 10 min at RT.

### Virus titration.

The extracellular virus titer was determined for supernatants from T lymphocytes inoculated with cell-free virus. Virus titers were assessed by a 50% tissue culture infective dose assay using RK13 cells. The 50% endpoint was calculated according to the method of Reed and Muench (90). T lymphocytes were resuspended in freshly added complete medium, followed by a freeze-thaw cycle, to analyze the intracellular virus titer.

### Cocultivation assays. (i) Cocultivation assay with EHV1-inoculated T lymphocytes and RK13 cells.

To detect and quantify EHV1-producing T lymphocytes, a cocultivation assay was performed with EHV1-inoculated T lymphocytes and the RK13 cell line. This assay was carried out as described previously by van der Meulen et al. (8). Briefly, mock- or EHV1-inoculated T lymphocytes were collected at 9 hpi. T lymphocytes were treated with citrate buffer for 1 min at RT. Cells were rinsed three times to remove redundant citrate buffer and then resuspended in 200 μl complete medium. Tenfold serial dilutions were made, and 200 μl of each dilution was added to RK13 monolayers in 24-well tissue culture plates. In parallel, citrate buffer-washed EHV1-inoculated T lymphocytes (9 hpi) were preincubated for 1 h at 37°C with 0, 0.1, 1, or 10 μM sodium orthovanadate (Na_3_VO_4_; Sigma-Aldrich), and this was maintained during cocultivation with RK13 cell monolayers. Inorganic vanadate (V_i_) is a potent inhibitor of dynein ATPase that functions by noncovalent trapping of the single ADP in the dynein-ADP-P_i_-complex by replacing P_i_ to form a stable dynein-ADP-V_i_ complex that acts as a dead-end kinetic block (50). As a positive control for the V_i_-mediated disruption of dynein activity, we monitored the cellular localization of the mannose 6-phosphate receptor (cf. indirect immunofluorescence staining) (91, 92). T lymphocytes cocultured with RK13 cell monolayers were overlaid with 0.94% carboxymethyl cellulose (CMC) medium (Sigma-Aldrich) prepared in 2× RPMI medium and supplemented with 4 U ml^−1^ hIL-2 and 50 mM β-ME and then were centrifuged at 800 × *g* for 30 min at 18°C. Cells were further cocultured for 48 h at 37°C and 5% CO_2_. In parallel, a viral plaque assay with cell-free virus diluted in medium containing V_i_ and RK13 cells was carried out to exclude direct V_i_ effects on viral replication. Briefly, RK13 cells were (mock) inoculated with the abortigenic 97P70 or neurovirulent 03P37 strain at an MOI of 0.1 in the presence of 0, 0.1, 1, or 10 μM V_i_ for 1 h at 37°C. Subsequently, cells were washed in citrate buffer (pH 3) for 1 min at RT. Cells were covered with 0.99% CMC containing corresponding concentrations of V_i_ and cultured for 24 h at 37°C. All cell monolayers were fixed with 10% formaldehyde and stained with 5% crystal violet, and the number of plaques was counted. The percentage of infected cells producing infectious EHV1 was calculated based on the number of plaques counted and the number of EHV1-positive cells seeded per well. Three independent experiments were performed. Concurrently, EHV1-inoculated T lymphocytes were inoculated with EHV1 and cultivated in the presence or absence of 400 μM phosphonoacetic acid (PAA) (Sigma-Aldrich). PAA specifically inhibits the activity of the herpesvirus DNA polymerase *in vitro* (93). Cells were collected at 9 hpi, washed with citrate buffer, and added to RK13 cell monolayers cultivated on inserts in 24-well tissue culture plates. After 2 h of cocultivation, RK13 cells and adherent T lymphocytes were fixed with either 100% methanol at −20°C for 20 min, to visualize intracellular (viral) proteins (immediate early proteins [IEP] and MTOC), or 2% PFA for 10 min at RT, to visualize adhesion molecules (active lymphocyte function-associated antigen 1 [LFA1]) on the cellular plasma membrane, followed by permeabilization in 0.1% Triton X-100 to stain viral proteins (IEP). Cells were stored at −20°C (methanol fixation) or 4°C (PFA fixation) until further processing.

### (ii) Cocultivation assay with EHV1-inoculated T lymphocytes and EC.

To analyze whether mock- or EHV1-inoculated T lymphocytes transfer infectious virus to EC, equine venous EC and EHV1-inoculated T lymphocytes were cocultured. At 12 hpi, EHV1-inoculated T lymphocytes were treated with citrate buffer as described above and then incubated with equine EC monolayers cultured in Lab-Tek II chamber slides with eight compartments (VWR) at a ratio of 2:1. The chamber slides were incubated for 36 h at 37°C and 5% CO_2_ on a rocking platform at 10 rpm.

### Indirect immunofluorescence staining. (i) Kinetics of viral protein expression.

At 1, 3, 6, 9, 12, and 24 hpi, EHV1-inoculated T lymphocytes were fixed in 1% PFA for 10 min at RT and washed two times with DPBS. Cells were prepared for immunofluorescence staining using cytospin centrifugation (7 min, 700 rpm). Next, cells were permeabilized with 0.1% Triton X-100 for 2 min at RT. Cells were incubated for 1 h at 37°C with a rabbit polyclonal antibody (pAb) against IEP (1:1,000) (94, 95) to visualize IEP and with a mouse monoclonal antibody (MAb) against either gB (clone 3F6) (IgG2a; 1:1,000) or gC (clone 14H7) (IgG2a; 1:1,000) to visualize the expression of late viral proteins. The IEP-specific antibody was kindly provided by D. J. O’Callaghan (USA). The gB and gC antibodies were kindly provided by U. Balasuriya (USA). Subsequently, cells were incubated for 50 min at 37°C with goat anti-rabbit IgG–FITC (1:100) or goat anti-mouse IgG–Texas Red (TR) (1:100) (Molecular Probes). All antibodies were diluted in DPBS. Cell nuclei were counterstained with Hoechst 33342 dye (10 μg ml^−1^) (Molecular Probes).

### (ii) Expression of viral proteins on the cell surface.

To analyze whether abortigenic and neurovirulent EHV1-infected T lymphocytes express viral proteins on the cell surface, double immunofluorescence staining was performed. At 9 hpi, T lymphocytes were fixed in 1% PFA, followed by cytospin centrifugation. The nonspecific binding sites (e.g., equine IgG receptor) were first blocked by incubation with 10% negative horse serum, obtained during a previous *in vivo* study by Vairo et al. (96), for 45 min at 37°C. Next, T lymphocytes were incubated with avidin and biotin (Thermo Fisher Scientific) to reduce nonspecific binding for 15 min at 37°C. To label viral proteins on the cell surface, a biotinylated horse anti-EHV1 pAb (1:20) (81) was used for 1 h at 37°C. Next, cells were permeabilized with 0.1% Triton X-100 for 2 min at RT, followed by incubation with a rabbit pAb against IEP (1:1,000) to visualize IEP expression. Subsequently, cells were incubated for 50 min at 37°C with streptavidin-FITC (1:100) and goat anti-rabbit IgG–TR (1:100) (Molecular Probes). Nuclei were counterstained with Hoechst 33342 dye (10 μg ml^−1^).

### (iii) Cell-to-cell viral transfer to T lymphocytes.

To determine viral transmission from EHV1-inoculated RK13 cells or EREC to T lymphocytes, double immunofluorescence staining was carried out. RK13 cells and EREC with potential bound T lymphocytes were incubated for 1 h at 37°C with a rabbit anti-IEP pAb (1:1,000) to visualize IEP and a mouse MAb against CD3 (IgG1; 1:50) to visualize T lymphocytes. Subsequently, cells were incubated for 50 min at 37°C with goat anti-rabbit IgG–TR (1:100) and goat anti-mouse IgG1–FITC (1:100) (Molecular Probes). Nuclei were counterstained with Hoechst 33342 dye (10 μg ml^−1^).

Triple immunofluorescence staining was performed to visualize viral reactivation in the EHV1-inoculated monocytic cells and viral transfer between EHV1-inoculated monocytic cells and T lymphocytes. Adherent cells and cytospin-centrifuged nonadherent cells were incubated with a rabbit pAb against IEP (1:1,000) to visualize IEP, a biotinylated pAb against EHV1 (1:20) (8) to visualize late viral proteins, and a mouse MAb against CD3 (IgG1; 1:50) to stain T lymphocytes. Next, cells were incubated for 50 min at 37°C with goat anti-rabbit–TR (1:100), streptavidin-FITC (1:200), and goat anti-mouse IgG1–Alexa Fluor 647 (1:100) (Molecular Probes). Nuclei were counterstained with Hoechst 33342 dye (10 μg ml^−1^).

### (iv) Viral transfer from T lymphocytes to target cells.

Double immunofluorescence staining was performed to visualize the polarization of EHV1-inoculated T lymphocytes upon adhesion to RK13 cells and EC. All T lymphocytes bound to the RK13 cells or EC were fixed with methanol and incubated with a rabbit pAb against IEP (1:1,000) to visualize IEP and a mouse MAb against gamma tubulin (IgG1; 1:50) (clone TU-30; Thermo Fisher) to visualize the cellular centrosome (MTOC). In parallel, T lymphocytes cocultured with the target cells were fixed with 2% PFA for 10 min at RT. Next, cells were incubated with a mouse MAb against CD18 (IgG1; 1:50) (clone MEM48; Invitrogen) to visualize the membrane expression of the integrin beta 2 subunit (active LFA1). Next, cells were permeabilized with 0.1% Triton X-100 for 2 min at RT, followed by incubation with a rabbit pAb against IEP (1:1,000) to visualize IEP expression. Subsequently, all cells were incubated for 50 min at 37°C with goat anti-rabbit–TR (1:100) and goat anti-mouse IgG1–FITC (1:100).

### (v) Cell markers.

***(a) CD4/CD8.*** The expression of CD4 and CD8 in the CD4/CD8 positively selected T lymphocytes was determined by immunofluorescence staining of cytospin-centrifuged T lymphocytes. Cells were incubated for 1 h at 37°C with a rabbit pAb against IEP (1:1,000) to visualize IEP and a mouse MAb against either CD4 (IgG1; 1:50) or CD8α (IgG3; 1:50) (Monoclonal Antibody Center, USA). Subsequently, cells were incubated for 50 min at 37°C with goat anti-rabbit IgG–TR (1:100) and goat anti-rabbit IgG–FITC (1:100) (Molecular Probes). Nuclei were counterstained with Hoechst 33342 dye (10 μg ml^−1^).

**(b) IL-2 receptor.** Upon T cell activation, the IL-2 receptor (IL-2R) was visualized by double immunofluorescence staining of cytospin-centrifuged T lymphocytes. Cells were incubated for 1 h at 37°C with a rabbit pAb against the IL-2 receptor (IgG; 1:50) (Biorbyt Ltd., United Kingdom) and a biotinylated pAb against EHV1 (1:20). Next, cells were incubated for 50 min at 37°C with goat anti-rabbit IgG–FITC (1:100) and streptavidin-TR (1:200) (Molecular Probes). Nuclei were counterstained with Hoechst 33342 dye (10 μg ml^−1^).

**(c) M6-PR.** Primary T lymphocytes or RK13 cells were incubated with 0 or 10 μM vanadate (V_i_) for 5 h prior to fixation (100% methanol, −20°C, 20 min). Cells were incubated for 1 h at 37°C with a MAb against the mannose 6-phosphate receptor (M6-PR) (IgG2a; 1:50) (clone 2G11; Abcam) to visualize M6-PR. Subsequently, cells were incubated for 50 min at 37°C with goat anti-mouse IgG–FITC (1:100) (Molecular Probes). Nuclei were counterstained with Hoechst 33342 dye (10 μg ml^−1^).

### Confocal microscopy.

Immunofluorescence staining of all cells was analyzed by confocal microscopy (Leica TCS SP2 laser scanning spectral confocal system; Leica Microsystems). A Gre-Ne 543-nm laser was used to excite Texas Red fluorochromes, an argon 488-nm laser to excite FITC fluorochromes, and a HeNe 594-nm laser to excite Alexa Fluor 647 fluorochromes.

### TEM.

To demonstrate viral transfer from EHV1-inoculated T lymphocytes to RK13 cells, we cultured T lymphocytes in complete medium at a concentration of 500,000 cells per well in a 24-well plate, followed by (mock) inoculation at an MOI of 5 for 1 h at 37°C. At 9 hpi, T lymphocytes were collected, washed in citrate buffer for 1 min at RT, and resuspended in complete medium supplemented with 5% HHS. The EHV1-inoculated T lymphocytes were cocultured with the RK13 cell monolayer, cultured on inserts (Electron Microscopy Sciences). At 2 h of cocultivation, nonadherent T lymphocytes were removed, and RK13 cells with adherent T lymphocytes were fixed for 1 h at RT with Karnovsky fixative as described above.

All samples were postfixed for 1 h with 1% OsO_4_ at RT. Next, cells were contrast stained *en bloc* for 1 h with 1% uranyl acetate, followed by dehydration steps in ascending grades of ethanol, and embedded in Spurr resin (100%) (Spurr low-viscosity embedding kit; EMS). After polymerization, the specimens were trimmed with a Leica EM Trim S4E type 702601 instrument. First, the blocks were sectioned into semithin sections (1 μm) on a Leica ultramicrotome (EM UC6) with Histojumbo knives and then stained with toluidine blue to select the most suitable areas for ultrathin sectioning. Next, ultrathin sections (±80 nm) were made with a Leica ultramicrotome (EM UC6) by use of a diamond knife (Diatome Ultra 45°; 2.5 mm) and collected on Formvar-coated copper grids (Formvar solution; EMS). Sections were contrast stained with lead citrate for 10 min. All sections were examined with a JEM-1400 Plus transmission electron microscope (JEOL, Benelux).

### Analysis of MTOC polarization.

To quantify MTOC polarization, EHV1-positive T lymphocytes attached to a single RK13 or endothelial cell were analyzed. Adherent T cells were scored as described by Pulecio et al. (97). Briefly, the ratio between the T-lymphocyte diameter and the distance of the MTOC to the synapse was calculated. Adherent T cells were considered polarized when this ratio was <0.3. Ten randomly selected conjugates were analyzed for each condition.

### Statistical analyses.

Data analyzed for statistical significance were subjected to a multiple-way analysis of variance (ANOVA). The Scheffé test was used as a *post hoc* test. If the assumption of equal variables was not fulfilled with Levene’s test, the data were log transformed prior to ANOVA. Normality of the residuals was verified by use of the Shapiro-Wilk test. The Kruskal-Wallis test followed by Mann-Whitney’s *post hoc* test was performed when variables remained unequal or when normality was not achieved after log transformation. Differences in results with *P* values of <0.05 were considered statistically significant. The data shown represent means plus standard deviations (SD) for independent experiments. Data were statistically evaluated with IBM SPSS Statistics for Windows, version 24.0 (IBM Corp., Armonck, NY, USA).
